# Approach to Interpreting Common Laboratory Pathology Tests in Transgender Individuals

**DOI:** 10.1210/clinem/dgaa546

**Published:** 2020-08-18

**Authors:** Ada S Cheung, Hui Yin Lim, Teddy Cook, Sav Zwickl, Ariel Ginger, Cherie Chiang, Jeffrey D Zajac

**Affiliations:** 1 Trans Health Research group, Department of Medicine (Austin Health), The University of Melbourne, Victoria, Australia; 2 Department of Endocrinology, Austin Health, Heidelberg, Victoria, Australia; 3 Diagnostics Haematology, Northern Pathology Victoria, Northern Health, Victoria, Australia; 4 ACON Health, Surry Hills, New South Wales, Australia; 5 Department of Diabetes and Endocrinology, Royal Melbourne Hospital, Victoria, Australia; 6 Department of Pathology, Royal Melbourne Hospital, Victoria, Australia

**Keywords:** transgender persons, pathology, biochemistry, troponin, kidney function tests

## Abstract

**Context:**

As the number of transgender (trans) people (including those who are binary and/or nonbinary identified) seeking gender-affirming hormone therapy rises, endocrinologists are increasingly asked to assist with interpretation of laboratory tests. Many common laboratory tests such as hemoglobin, iron studies, cardiac troponin, and creatinine are affected by sex steroids or body size. We seek to provide a summary of the impact of feminizing and masculinizing hormone therapy on common laboratory tests and an approach to interpretation.

**Cases:**

Case scenarios discussed include 1) hemoglobin and hematocrit in a nonbinary person undergoing masculinizing hormone therapy; 2) estimation of glomerular filtration rate in a trans woman at risk of contrast-induced nephropathy; 3) prostate-specific antigen (PSA) in a trans woman; and 4) chest pain in a trans man with a cardiac troponin concentration between the reported male and female reference ranges.

**Conclusions:**

The influence of exogenous gender-affirming hormone therapy on fat and muscle distribution and other physiological changes determines interpretation of laboratory tests that have sex-specific differences. In addition to affirmative practice to ensure a patient’s name, gender, and pronoun are used appropriately, we propose that once individuals have commenced gender-affirming hormone therapy, the reference range of the affirmed gender be reported (and specified by treating clinicians) except for PSA or cardiac troponin, which are dependent on organ size. While suggestions may be challenging to implement, they also represent an opportunity to lead best practice to improve the quality of care and experiences of healthcare for all trans people.

As the number of transgender (trans) people (including those who are binary and/or nonbinary identified) seeking gender-affirming hormone therapy rises in society (1), endocrinologists are increasingly asked to assist with interpretation of laboratory test results. Many common laboratory tests such as hemoglobin, iron studies, cardiac troponin, and creatinine are affected by sex steroids or body size determined during pubertal growth and as such, have specific reference ranges (2). The difference between sex-specific reference ranges for these analytes are most marked during puberty, as the pubertal spikes of testosterone occur later in those presumed male at birth (3). We seek to provide a summary of the impact of feminizing and masculinizing hormone therapy on common clinical laboratory tests and an approach to interpretation.

Trans people are individuals whose gender is different than that presumed for them at birth and includes people with a binary and/or nonbinary gender identity. Estimates worldwide suggest that from 0.6% to 1.2% of the general population are trans or gender-diverse (4, 5). Due to often intense feelings of incongruence between one’s gender and body (termed dysphoria), many trans individuals undergo gender-affirming hormone therapy to align their physical characteristics with their gender to improve psychological, social, and cultural functioning (6). Masculinizing hormone therapy is typically testosterone alone, which induces significant gains in muscle mass, decrease in fat mass, and fat redistribution, as well as deepening of the voice and facial and body hair growth (7, 8). Feminizing hormone therapy is usually estradiol and an anti-androgen, such as spironolactone or cyproterone acetate, which will induce body fat redistribution to a more gynoid pattern with increases in fat mass and decreases in muscle mass, as well as skin softening, decreased libido, and breast growth (7, 9). Target sex-steroid reference ranges on gender-affirming hormone therapy are generally the normal reference ranges of an individual’s affirmed gender, rather than the gender presumed for them at birth (10-12). Notably, people with nonbinary gender identities who seek medical affirmation may undergo partial masculinization with low-dose testosterone therapy to target sex-steroid reference ranges that may be lower than the typical “male” reference range (or conversely, partial feminization). Alternatively, some may desire a slower rate of physical change and hence use low-dose or microdose gender-affirming hormone therapy (13). While many trans people will seek to update their legal name and gender marker to align with their identity, costs to do so are prohibitive for many (14). Using a former pronoun, gender marker, or name, even if still listed on identity documents, can cause intense distress and lead to, or exacerbate, dysphoria for an individual (15). Due to current limitations of electronic medical records, laboratory information systems and health professionals’ understanding of gender identity, interactions with laboratory services and healthcare providers can often magnify severe distress and affect the mental health of an individual. This may result in the individual’s reluctance to return to care.

While there are publications describing the effects of feminizing or masculinizing hormone therapy on gender-specific laboratory tests, there is a lack of long-term outcome data on the safety of using a particular reference range. As such, we base most of this discussion on physiological principles. Furthermore, it is unclear if; 1) the reference ranges are a direct switch to the affirmed gender identity for analytes which are immediately affected by sex hormones (eg, growth hormone); 2) whether analytes which are determined during pubertal growth but are not affected by sex hormones in later life (eg, troponin, which reflects cardiac size) should continue with the reference range of the gender presumed at birth or; 3) whether trans-specific reference ranges are required for a period after gender-affirming hormones are commenced to monitor analytes that are gradually affected by sex hormones (eg, creatinine which reflects muscle mass). The pivotal question is how we can have a “one size fits all” solution to cater for a heterogenous group who have sex-steroid concentrations and resultant body composition changes that increase or decrease at different velocities and magnitudes?

## Sex-steroid concentrations

While guidelines recommend targeting sex-steroid reference ranges of the affirmed gender for people seeking full masculinization or feminization, this is based predominantly upon expert opinion and in some instances, there is debate whether measurement of sex steroids are indeed clinically useful at all. One example of this is measurement of serum estradiol concentrations in those on feminizing hormone therapy, with some clinicians adjusting therapy to estradiol concentrations whereas others adjust therapy based on clinical response (10-12). No studies have evaluated the optimal estradiol concentration for feminization in people presumed male at birth. In clinical practice, several clinical cohorts of trans individuals in Europe, United States, and Australia have reported the sex-steroid concentrations achieved in specialized gender clinics. These studies report that individuals undergoing feminizing hormone therapy for at least 6 months typically achieve estradiol concentrations in the range of 211 to 400 pmol/L (57-109 pg/mL) with testosterone concentrations of 2 to 4 nmol/L (0.57-1.15 ng/mL) (7, 16-19). These changes in sex-steroid concentrations induce some shifts from lean mass to fat mass, which impacts laboratory tests such as creatinine (20).

For trans people presumed female at birth who are using masculinizing hormone therapy, testosterone concentrations rise from the female reference range of <2 nmol/L (<0.57 ng/mL) up to the male reference range of 10 to 35 nmol/L (2.88-10.09 ng/mL) (7, 18, 21). It has been noted that despite masculinizing hormone therapy increasing testosterone concentrations, serum estradiol concentrations do not fall dramatically. A retrospective chart review in a US gender-affirmation clinic, found that mean estradiol only decreased by 26 pmol/L, from 217 to 191 pmol/L (59-52 pg/mL) after 6 months on masculinizing hormone therapy (16). Consistent with this, a European cohort observed that after commencing testosterone therapy, serum estradiol concentrations in trans men decreased by approximately 50 to 60 pmol/L (13.6 to 17.1 pg/mL) from baseline (18, 22). In the setting of high testosterone concentrations, the estradiol concentration per se does not affect masculinization, with significant gains in muscle mass and strength of approximately 10% and loss of fat mass (8). In addition to muscle mass gains, high testosterone concentrations also significantly impact upon hematopoiesis and interpretation of hematology laboratory tests.

## Case 1

*A 28-year-old nonbinary individual presumed female at birth has recently commenced full masculinizing hormone therapy with transdermal testosterone gel. You receive a referral from their primary care physician concerned about polycythemia. Their hemoglobin is 168 g/L with hematocrit 0.49, which has been flagged in the laboratory report as high (reported with female reference range of 115-155g/L and 0.33-0.45 relative to the male reference interval of 120-170g/L and 0.36-0.50).*

### Hematology

Androgens are known to stimulate erythropoiesis while the impact of estrogens are not as well understood. In trans people who have been on established and full-dose feminizing hormone therapy (estradiol and anti-androgen) for at least 6 months, there is a significant decrease in hemoglobin, hematocrit, and red blood cell count to the female reference range (16, 23-25). Conversely after 6 months of masculinizing testosterone therapy, trans people demonstrate an increase in hemoglobin, hematocrit, and red blood cell count to the male reference range (16, 20, 23, 26). Serum hematocrit in the range of the affirmed gender is evident from 3 months after commencing gender-affirming hormone therapy (27). Of note, there are association studies suggesting higher hematocrit is associated with a higher risk of cardiovascular disease (28, 29). This is probably a consideration for people using masculinizing hormone therapy, more so than those using feminizing hormone therapy. As smoking may additionally increase hematocrit, smoking cessation should be emphasized in those with elevated hematocrit. While the long-term cardiovascular implications of using a different reference range for hemoglobin or hematocrit are unclear in general, reference ranges of the affirmed gender should be used. Female reference ranges should be used for someone taking gender-affirming feminizing hormone therapy and male reference ranges should be used for people using masculinizing hormone therapy.

In trans women, there is a small statistically significant but clinically insignificant rise in platelet count (which remains within the normal reference range) shown in several cohort studies after 6 to 12 months of feminizing hormone treatment (16, 19), while white blood cells do not change significantly. No apparent changes are observed in either platelet count or white blood cells with masculinizing hormone therapy (16, 19).

*Case 1 outlines a nonbinary individual presumed female at birth receiving full-dose masculinizing hormone therapy. In this case, the male reference range for hemoglobin and hematocrit would be most appropriate and this should be shared with the nonbinary individual so they are aware and can expect to be misgendered when reviewing their own results. As such, their hemoglobin of 168 g/L and hematocrit of 0.49 would fall within the expected reference range and no change in management needs to occur.*

### Iron studies

Reference ranges for serum ferritin, a common indicator of body iron status, vary depending on age and sex (30). Ferritin reference ranges are typically lowest in premenopausal people presumed female at birth, followed by postmenopausal people and are highest in people presumed male at birth, with lower limits of the female reference range approximately 10 to 20 ug/L below that of the male reference range (30 ug/L) (31). This may be partially attributed to increased iron utilization in menstruating individuals resulting in lower ferritin, as well as a multitude of factors that have been shown to impact upon adult serum ferritin levels including age, body mass index, waist to hip ratio, and liver function (30, 32). Animal studies suggest that iron is distributed differently in males and females associated with differences in hepatic hepcidin expression rather than sex-steroid concentrations (33, 34). No studies have evaluated whether ferritin or other iron indicators change with gender-affirming hormone therapy.

From a practical perspective, the main reason to evaluate for iron deficiency is anemia. In individuals who have a ferritin below the “male” reference range, we suggest interpreting the iron studies in the context of red cell indices such as mean corpuscular volume and mean corpuscular hemoglobin concentration to guide management rather than on the use of gender-affirming hormone therapy. If the trans individual is menstruating or pregnant, it would be most practical to use the premenopausal female reference range for interpretation of iron studies.

For evaluation of possible iron overload, in situations of borderline results which fall between the female and male reference ranges, relying on the absolute ferritin level or transferrin saturation will be difficult. It is pertinent to assess for concurrent inflammatory disease, liver disease, or iron overload states, such as hemochromatosis, which may further guide clinical management.

## Case 2

*A cardiologist calls as they are planning a coronary angiogram for a 68-year-old trans woman and are concerned because the estimated glomerular filtration rate (eGFR) is unknown. They are uncertain how to risk stratify her for potential contrast-induced nephropathy. She has a history of longstanding hypertension and hypercholesterolemia, vaginoplasty, and has been on various formulations of estradiol therapy for over 20 years. On review of her investigations, her serum creatinine is 109 umol/L (1.23 mg/dL) but her eGFR has not been reported for the last 18 months. Laboratory providers cannot report eGFR if a male or female marker is not provided on the request form, as this is required along with age to estimate eGFR. Using the Chronic Kidney Disease Epidemiology Collaboration (CKD-EPI) formula, if classified as female, the eGFR would be 45 mL/min/1.73m* ^*2*^ *classed as Stage 3 chronic kidney disease and would meet the guidelines for intravenous hydration prior to procedure. However, if classified male, the patient would have an eGFR of 60 mL/min/1.73m* ^*2*^ *which would be classed as Stage 2 chronic kidney disease and would not require prehydration. Which is the most appropriate eGFR to use?*

### Renal function

Accurately assessing renal function is essential for not only assessment of renal diseases, but also clinical situations that may potentially affect renal function (such as diabetes or radioiodine contrast administration) as well as considerations for medication dosing of renally cleared drugs. The most commonly used marker of renal function in clinical pathology laboratories is eGFR, which is calculated based upon an individual’s serum creatinine level, age, and sex (35). Typically, people presumed male at birth have a higher eGFR than people presumed female at birth at the same level of serum creatinine because the formula assumes a higher muscle mass in men contributing to the serum creatinine independent of renal function. The difference between these groups (given the same age and weight) is more marked at higher levels (with a difference of approximately 30 when eGFR >90 mL/min/1.73m^2^), becoming much more similar as eGFR declines (difference of approximately 4 when eGFR <30 mL/min/1.73m^2^). In clinical situations where accurate assessment of renal function is necessary, such as in the transplant setting, it may be more appropriate to use 24-hour urine creatinine clearance, urinary inulin clearance (36), or serum cystatin c levels, which are less affected by sex and not affected by muscle mass in contrast to serum creatinine (37). Inulin clearance and cystatin c are more expensive and less readily available. Creatinine clearance can be calculated on paired 24-hour urine and serum creatinine concentration and is independent of muscle mass and sex steroids. This can provide a baseline estimation for renal function and cumulative serum creatinine results can then be used to monitor decline in renal function with aging.

From a practical perspective, laboratory reports will need to make an assessment on how to report the eGFR for trans individuals. For individuals receiving masculinizing or feminizing hormone therapy, changes in body composition appear to be maximal in the early period after commencement, evident within the first 3 months of treatment (38, 39). For those receiving masculinizing hormone therapy with testosterone, given higher muscle mass and lower fat mass compared to females, the male CKD-EPI formula would be more appropriate. Conversely if a person has been on feminizing hormone therapy, which typically induces gain in fat and decrease in muscle mass from 3 months of use, then the female equations should be used. It would be a challenging task to expect pathology laboratories to provide the “right” eGFR given limited access to clinical information.

We recommend that the treating clinician specify the sex-specific reference interval desired for reporting on the laboratory request (ie, female for a trans person using feminizing hormone therapy). Using current laboratory information systems, the gender marker can be used as a field to specify the reference range desired for reporting. While the binary female or male gender may not necessarily reflect the individual’s gender, this will allow for the appropriate reference range to be reported and the trans patient informed so they can prepare to be misgendered. For laboratory providers, if the gender marker is unknown, then treating clinicians should be contacted to specify the sex-specific reference interval desired.

*For the trans woman described in Case 2 who was on longstanding feminizing hormone therapy with female body composition, the female reference range for renal function would be most appropriate triggering appropriate renoprotection prior to administration of radioiodine contrast for her angiogram. From a harm reduction approach, given the absence of data in the field, if either the male or female calculated eGFR suggests renoprotective strategies, then this can be implemented. A 24-hour urine creatinine clearance can also be performed to more accurately assess renal function.*

## Case 3

*A 70-year-old trans woman who had been on feminizing hormone therapy for 6 months had a PSA performed as part of a routine health check. She was taking transdermal estradiol 100mcg/24hr patches twice weekly and cyproterone acetate 12.5mg daily. Her total testosterone was 1.5 nmol/L (43 ng/dl) and PSA was 2 ng/mL. She had mild lower urinary tract symptoms with reduced urinary flow over a number of years but had no family history of prostate cancer. How should she be managed?*

### Prostate-specific antigen

There are no studies examining the effect of feminizing hormone therapy on PSA. It is known that androgen deprivation as part of feminizing hormone therapy is associated with a substantially lower risk for prostate cancer than the general male population (40). All published case reports of prostate cancer in trans people using feminizing hormone therapy have had histology showing high risk adenocarcinoma with PSA concentrations at diagnosis ranging from 5 to 1722 ng/mL (ng/mL equivalent to ug/L) (40, 41). Physiologically, in the setting of androgen deprivation in people with a prostate gland, it would be expected that PSA should be lower than the age-specific reference interval. There is insufficient data to recommend a specific cutoff for trans people using feminizing hormone therapy. Individualized decisions based upon clinical history and examination should inform need for serial monitoring for PSA velocity or imaging.

*Case 3 had a digital rectal examination which showed a smooth but mildly enlarged prostate gland. She had an ultrasound of her prostate which showed a mildly enlarged prostate volume of 35 mL. Repeat PSA monitoring revealed progressive lowering of her PSA concentration with ongoing feminizing hormone therapy and an improvement in her urinary flow.*

## Case 4

*A 49-year-old trans man who had been on testosterone therapy for 10 years presented to the emergency department with central chest pain. His high-sensitivity cardiac troponin was 24 ng/L (female reference range <16 ng/L, male reference range <26 ng/L). How should he be managed?*

### High-sensitivity cardiac troponin

Cardiac troponin is released from damaged cardiomyocytes and is one of the most common biomarkers used in the prediction of myocardial infarction. There is considerable debate regarding the use of sex-specific reference ranges for high-sensitivity cardiac troponin (hs-cTn), as there is uncertainty whether the use of sex-specific reference limits impact upon clinical management or outcome prediction (42). However, as upper reference limits based on sex-specific 99th percentiles for hs-cTn are subtly higher for people recorded as males than those recorded females in population studies (43), use of sex-specific cutoffs for hs-cTn assays have been endorsed by the International Federation of Clinical Chemistry and Laboratory Medicine (44). The difference has been attributed to people presumed male at birth having a larger cardiac mass as well as subclinical coronary artery disease (45). No studies have been performed to examine cardiac mass changes that may occur with masculinizing hormone therapy in people presumed female at birth. There are however data in polycystic ovary syndrome in which high testosterone concentrations are a clinical feature (albeit far lower than testosterone concentrations seen in transgender men). Polycystic ovary syndrome has been associated with higher left ventricular mass index and larger left atrial diameter over 5 years of follow-up, even after adjustment for blood pressure, body mass index, glucose, and lipids (46). Large population-based studies have also shown that left ventricular mass correlates with body weight, lean body mass, and fat mass (47). There is currently insufficient data to draw an inference regarding the appropriate reference range in people using gender-affirming hormone therapy, and emphasis must be placed on clinical history, electrocardiogram (ECG) changes, and serial trajectory of hs-cTn levels if the hs-cTn falls in between the male and female-specific reference ranges.

*Despite the fact that Case 4 had been on established testosterone therapy for 10 years with resultant male body composition, there is insufficient data to suggest that cardiac remodeling or change in cardiac size occurs* *with high (or low) testosterone concentrations. Despite the risk of being oversensitive, in order to minimize the risk of missing an acute coronary event, we suggest that the reference range of the sex presumed at birth (female) should be used to interpret hs-cTn, provided the patient is informed of this rationale in addition to monitoring with serial troponin to ensure there is no rise. Case 3’s subsequent hs-cTn was elevated above the male reference range and his ECG revealed anterior ST-segment depression consistent with acute coronary syndrome.*

## Recommendations

Given that changes in sex-steroid concentrations, body composition, and common laboratory values begin to occur within 3 months of gender-affirming hormone therapy, we recommend that once an individual has commenced gender-affirming hormone therapy, the reference range of the individual’s affirmed gender (either female or male—see below for nonbinary recommendations) should be used for tests with sex-specific reference ranges, except for tests dependent on organ size for which the reference range for the sex presumed at birth should be used (cardiac troponin, PSA). An overview of recommended reference ranges for common laboratory tests is provided in the Table 1. Individualized interpretation and decision-making will still need to occur, particularly for individuals early in the course of gender-affirming therapy and for individuals on low doses of gender-affirming hormones, nonstandard regimens, or concurrent medical conditions.

**Table 1.** Recommendations for Laboratory Tests With Sex-Specific Reference Ranges in Trans People Using Gender-Affirming Hormone Therapy

Test	Recommended Reference Range for Interpretation		Comments
	Affirmed Gender	Presumed Sex at Birth	
Estradiol	✓		
Total Testosterone	✓		
Creatinine	✓		
Estimated GFR	✓		Alternatively, perform a 24-hour urine creatinine clearance.
Hemoglobin	✓		
Hematocrit	✓		
Iron studies	✓		Insufficient data. Premenopausal female reference range should be used for menstruating or pregnant individuals regardless of gender.
Electrolytes	✓		No sex-specific reference ranges. Minor changes in sodium observed in small retrospective uncontrolled studies; sodium reduced with feminizing hormone therapy and increased with masculinizing hormone therapy.
Liver function	✓		No sex-specific reference ranges. There is no clear evidence to suggest clinically significant changes occur with gender-affirming hormone therapy (16, 19, 23, 25, 48).
Lipid profile	✓		No sex-specific reference ranges. Masculinizing hormone therapy associated with decreases in HDL-c (19, 20, 23, 24, 49, 50). Feminizing hormone therapy associated with inconsistent lipid effects (19, 23-25, 51). If raised triglycerides observed, consider use of transdermal rather than oral estradiol formulations (52).
Prostate-specific antigen (PSA)		✓	Valid only for people with a prostate. The prostate remains in situ even after orchiectomy, vaginoplasty, or labioplasty surgery. PSA is expected to be low in the setting of low testosterone concentrations.
High-sensitivity cardiac troponin		✓	Cardiac troponin is based upon organ size, which is not expected to change with gender-affirming hormone therapy.

Note that consideration should be made as to the duration and dose of feminizing or masculinizing hormone therapy used in interpretation of laboratory tests.

In the absence of nonbinary reference ranges, for people who may be using low-dose masculinizing hormone therapy as part of nonbinary gender affirmation, the appropriate reference range is typically somewhere between the male and female reference ranges. This poses challenges for reporting, and from a practical perspective, we recommend that, similar to people using standard doses of gender-affirming hormones, for someone using masculinizing hormones, the male reference range be used, and for someone using feminizing hormones, the female reference range be used, except for laboratory tests dependent on organ size such as cardiac troponin. We acknowledge that there is no “one size fits all” and interpretation ideally should be individualized by the treating clinician based on the clinical information. Similarly, in the early period after newly commencing gender-affirming hormones, the optimal range should be individualized as the serum sex-steroid concentrations of the affirmed gender gradually increase to their target concentrations.

Treating clinicians should clearly specify on the laboratory request whether the female or male reference range should be reported. This may be easily satisfied within current constraints of laboratory information systems by specifying the corresponding gender marker for the patient on the laboratory request. In instances where the gender marker is unknown or not specified, clinicians should be contacted to obtain the desired reference interval to be reported (ie, the female reference range for someone on feminizing hormone therapy). Laboratories should always report a reference range to ensure that critical result notifications are triggered and appropriately actioned. Dual reporting of both male and female reference ranges is another potential option, however current laboratory information system barriers exist. Dual reporting and identifying a person as trans in the gender marker field has the potential to lead to confusion and potentially open the opportunity for discrimination, which is feared by many trans people.

The challenge for medical record systems and laboratory information systems will be ensuring that affirming practices are in place to provide quality care for trans people. While our recommendations propose a simplified approach for laboratories, this may pose challenges when more than one test and reference range is desired in an episode (such as renal function and cardiac troponin). Ideally, system fields that encompass legal name, preferred name, presumed gender at birth, actual gender, and pronoun, which is particularly relevant for treating clinicians and laboratory test collection staff, should be incorporated. Signage or posters indicating to patients the need to obtain sensitive information such as presumed gender at birth (which may induce dysphoria for some but is of clinical importance for tests such as cardiac troponin), may help convey a safe, affirming space for trans people.

## Conclusions

There is increasing visibility of trans individuals globally and it is likely that endocrinologists will be asked to interpret common laboratory results for people receiving gender-affirming hormone therapy. We propose that once individuals have commenced gender-affirming hormone therapy, that the reference range of the affirmed gender be reported, other than for PSA and cardiac troponin, which are dependent upon organ size. The influence of exogenous gender-affirming hormone therapy on fat and muscle distribution and other physiological changes determines the interpretation of most laboratory tests that have sex-specific differences. This vital piece of clinical information should be provided by clinicians to laboratory staff and the desired reference range can be reflected in current laboratory information systems by using the corresponding gender marker on the laboratory request. As the binary male or female marker may not always reflect a trans person’s gender, communication to explain the limitations are essential. While these suggestions may be challenging to implement, they also represent an opportunity to lead best practice to improve the quality of care and experiences of health and healthcare for all trans people.

## Abbreviations

CKD-EPI: Chronic Kidney Disease Epidemiology Collaboration
ECG: electrocardiogram
eGFR: estimated glomerular filtration rate
hs-cTn: high-sensitivity cardiac troponin
PSA: prostate-specific antigen

## Additional Information

***Disclosure Summary:*** The authors have nothing to disclose.

***Data Availability:*** Data sharing is not applicable to this article as no datasets were generated or analyzed during the current study.
